# Penta­kis(ethyl­enediammonium) tri-μ-sulfato-bis­[tris­ulfatocerate(IV)] trihydrate

**DOI:** 10.1107/S1600536810021811

**Published:** 2010-06-16

**Authors:** Nadia Jabeen, Saeed Ahmad, Ali Farooq Meer, Islam Ullah Khan, Seik Weng Ng

**Affiliations:** a Department of Chemistry, University of Engineering & Technology, Lahore 54000, Pakistan; b Department of Chemistry, Government College University, 54000 Lahore, Pakistan; c Department of Chemistry, University of Malaya, 50603 Kuala Lumpur, Malaysia

## Abstract

In the cerate(IV) anion of the title salt, (C_2_H_10_N_2_)_5_[Ce_2_(SO_4_)_9_]·3H_2_O, the two metal atoms are bridged by three sulfate units; each metal atom is itself chelated by other three sulfate units so that the metal atoms exist in nine-coordinate tricapped trigonal-prismatic geometries. The anions, cations and uncoordinated water mol­ecules are linked by O—H⋯O and N—H⋯O hydrogen bonds, forming a three-dimensional network. One of the five cations is disordered with respect to the ethyl­ene portion in a 1:1 ratio.

## Related literature

For the crystal structures of other ethyl­enediammonium sulfato­cerate(III) salts, see: Fu *et al.* (2005 ▶, 2006 ▶).
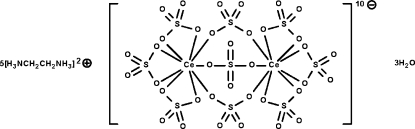


## Experimental

### 

#### Crystal data

(C_2_H_10_N_2_)_5_[Ce_2_(SO_4_)_9_]·3H_2_O
*M* *_r_* = 1509.43
Triclinic, 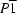

*a* = 11.6716 (2) Å
*b* = 13.3506 (2) Å
*c* = 15.4143 (2) Å
α = 83.8823 (5)°
β = 88.0962 (5)°
γ = 73.7832 (6)°
*V* = 2293.18 (6) Å^3^
*Z* = 2
Mo *K*α radiation
μ = 2.49 mm^−1^
*T* = 293 K
0.15 × 0.10 × 0.05 mm

#### Data collection

Bruker Kappa APEXII diffractometer
Absorption correction: multi-scan (*SADABS*; Sheldrick, 1996 ▶) *T*_min_ = 0.706, *T*_max_ = 0.885
39247 measured reflections
10407 independent reflections
9501 reflections with *I* > 2σ(*I*)
*R*_int_ = 0.032

#### Refinement

*R*[*F* ^2^ > 2σ(*F* ^2^)] = 0.029
*wR*(*F* ^2^) = 0.117
*S* = 1.25
10407 reflections
637 parameters
37 restraints
H-atom parameters constrained
Δρ_max_ = 1.80 e Å^−3^
Δρ_min_ = −1.56 e Å^−3^

### 

Data collection: *APEX2* (Bruker, 2009 ▶); cell refinement: *SAINT* (Bruker, 2009 ▶); data reduction: *SAINT*; program(s) used to solve structure: *SHELXS97* (Sheldrick, 2008 ▶); program(s) used to refine structure: *SHELXL97* (Sheldrick, 2008 ▶); molecular graphics: *X-SEED* (Barbour, 2001 ▶); software used to prepare material for publication: *publCIF* (Westrip, 2010 ▶).

## Supplementary Material

Crystal structure: contains datablocks global, I. DOI: 10.1107/S1600536810021811/xu2776sup1.cif

Structure factors: contains datablocks I. DOI: 10.1107/S1600536810021811/xu2776Isup2.hkl

Additional supplementary materials:  crystallographic information; 3D view; checkCIF report

## Figures and Tables

**Table 1:** Hydrogen-bond geometry (Å, °)

*D*—H⋯*A*	*D*—H	H⋯*A*	*D*⋯*A*	*D*—H⋯*A*
O1w—H11⋯O24^i^	0.84	2.02	2.803 (5)	156
O1w—H12⋯O28	0.84	1.96	2.786 (5)	169
O2w—H21⋯O16^ii^	0.84	1.94	2.754 (5)	162
O2w—H22⋯O7^iii^	0.84	1.99	2.828 (5)	173
O3w—H31⋯O9^iv^	0.84	2.47	3.153 (7)	139
N1—H1a⋯O10^i^	0.86	2.11	2.899 (5)	151
N1—H1b⋯O27	0.86	2.28	2.991 (6)	141
N1—H1c⋯O1w	0.86	2.09	2.891 (6)	154
N2—H2a⋯O28^ii^	0.86	2.02	2.863 (5)	165
N2—H2b⋯O8^iii^	0.86	2.16	2.769 (5)	128
N2—H2c⋯O29^ii^	0.86	2.01	2.849 (5)	163
N3—H3a⋯O1w	0.86	2.10	2.863 (7)	148
N3—H3b⋯O3w	0.86	1.93	2.753 (8)	160
N3—H3c⋯O26^ii^	0.86	2.20	3.048 (7)	167
N4—H4a⋯O2w^v^	0.86	1.95	2.760 (7)	157
N4—H4b⋯O36^ii^	0.86	2.16	2.843 (6)	136
N4—H4c⋯O6^iv^	0.86	2.37	3.119 (6)	145
N5—H5a⋯O1	0.86	2.27	3.029 (5)	148
N5—H5b⋯O20^vi^	0.86	2.09	2.794 (5)	138
N5—H5c⋯O16	0.86	2.00	2.763 (5)	147
N6—H6a⋯O12^vii^	0.86	2.21	2.837 (6)	130
N6—H6b⋯O1^vi^	0.86	2.46	3.241 (5)	152
N6—H6c⋯O31	0.86	1.99	2.826 (6)	165
N7—H7′a⋯O15	0.86	2.27	2.935 (6)	135
N8—H8a⋯O34^v^	0.86	2.21	2.957 (6)	145
N8—H8b⋯O23^iv^	0.86	2.23	2.840 (6)	128
N9—H9a⋯O35	0.86	2.40	2.992 (6)	126
N9—H9b⋯O24	0.86	2.10	2.846 (5)	145
N9—H9c⋯O19	0.86	2.06	2.895 (5)	165
N10—H10a⋯O11	0.86	2.04	2.879 (6)	165
N10—H10b⋯O30^viii^	0.86	2.39	3.176 (6)	153
N10—H10c⋯O19	0.86	2.05	2.884 (6)	164

Symmetry codes: (i) 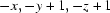; (ii) 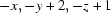; (iii) 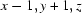; (iv) 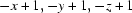; (v) 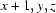; (vi) 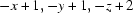; (vii) 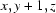; (viii) 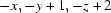.

**Fig. 1.**
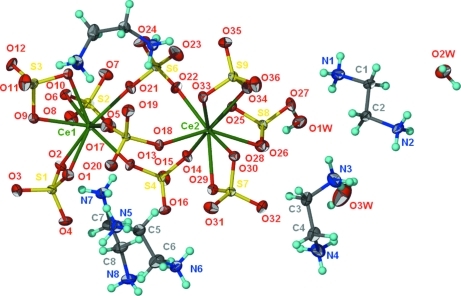
Thermal ellipsoid plot (Barbour, 2001) of (C10H10N2)52+ [Ce2(SO4)9]10-.3H2O at the 50% probability level; hydrogen atoms are drawn as spheres of arbitrary radius. The disorder in one of the cations is not shown.

**Fig. 2.**
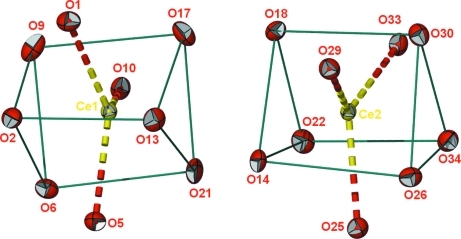
Tricapped trigonal prismatic geometries of the two cerium atoms.

## supplementary crystallographic information

### Comment

The reaction of cerium nitrate and ethylenediamine in concentrated sulfuric acid yields (C_2_H_10_N_2_)_3_[Ce_2_(SO_4_)_6_(H_2_O)_2_].4H_2_O, a compound adopting a layer structure (Fu *et al.*, 2005). Under hydrothemal conditions, (C_2_H_10_N_2_)_2_[Ce_2_(SO_4_)_5_(H_2_O)_2_], which adopts a chain structure, is obtained (Fu *et al.*, 2006). The two salts have the metal atom in the +3 oxidation state. Under less drastic conditions, a cerate(IV) salt results (Scheme I, Fig. 1). The cerate(IV) anion of the salt, (C_10_H_10_N_2_)_5_^2+^ [Ce_2_(SO_4_)_9_]^10-.^3H_2_O, has the two metal atoms bridged by three sulfate units; each metal atom itself is chelated by three sulfate units so that the metal atoms exist in nine-coordinate tricapped trigonal prismatic geometries (Fig. 2). The anions, cations and lattice water molecules are linked by O–H···O and N–H···O hydrogen bonds to form a three-dimensional network.

### Experimental

Ethylenediamine (0.33 ml, 5 mmol) dissolved in water (10 ml) was added to cerium(IV) sulfate tetrahydrate (0.40 g, 1 mmol) dissolved in water (20 ml). Concentrated sulfuric acid (4 drops) were added. The solution was filtered and set aside for the growth of the faint yellow crystals.

### Refinement

Carbon-bound H-atoms were placed in calculated positions (C–H 0.93 to 0.98 Å, N–H 0.86 Å) and were included in the refinement in the riding model approximation, with *U*(H) set to 1.2 to 1.5*U*(C,N).

The water H-atoms were placed in chemically sensible positions on the basis of hydrogen bonding but were not refined (O–H 0.84±0.01 Å).

One of the cations is disordered with respect to the ethylene portion. The N–C and C–C distances of the unprimed and primed atoms were restrained to within 0.01 Å of each other, and the temperature factors of the primed atoms were set to those of the unprimed ones. The anisotropic temperature factors were restrained to be nearly isotropic. The occupancy could not be refined, and was fixed as 1:1.

The final difference Fourier map had a peak at 2 Å from H10d and a hole in the vicinity of Ce2. Attempts to refine the peak as an oxygen atom led to an impossibly large temperature factor.

### Crystal data

**Table:**

(C_2_H_10_N_2_)_5_[Ce_2_(SO_4_)_9_]·3H_2_O	*Z* = 2
*M**_r_* = 1509.43	*F*(000) = 1516
Triclinic, *P*1	*D*_x_ = 2.186 Mg m^−^^3^
Hall symbol: -P 1	Mo *K*α radiation, λ = 0.71073 Å
*a* = 11.6716 (2) Å	Cell parameters from 9964 reflections
*b* = 13.3506 (2) Å	θ = 2.7–28.3°
*c* = 15.4143 (2) Å	µ = 2.49 mm^−^^1^
α = 83.8823 (5)°	*T* = 293 K
β = 88.0962 (5)°	Prism, yellow
γ = 73.7832 (6)°	0.15 × 0.10 × 0.05 mm
*V* = 2293.18 (6) Å^3^	

### Data collection

**Table:**

Bruker Kappa APEXII diffractometer	10407 independent reflections
Radiation source: fine-focus sealed tube	9501 reflections with *I* > 2σ(*I*)
graphite	*R*_int_ = 0.032
φ and ω scans	θ_max_ = 27.5°, θ_min_ = 1.3°
Absorption correction: multi-scan (*SADABS*; Sheldrick, 1996)	*h* = −15→14
*T*_min_ = 0.706, *T*_max_ = 0.885	*k* = −17→17
39247 measured reflections	*l* = −19→20

### Refinement

**Table:**

Refinement on *F*^2^	Primary atom site location: structure-invariant direct methods
Least-squares matrix: full	Secondary atom site location: difference Fourier map
*R*[*F*^2^ > 2σ(*F*^2^)] = 0.029	Hydrogen site location: inferred from neighbouring sites
*wR*(*F*^2^) = 0.117	H-atom parameters constrained
*S* = 1.25	*w* = 1/[σ^2^(*F*_o_^2^) + (0.063*P*)^2^ + 3.8392*P*] where *P* = (*F*_o_^2^ + 2*F*_c_^2^)/3
10407 reflections	(Δ/σ)_max_ = 0.001
637 parameters	Δρ_max_ = 1.80 e Å^−^^3^
37 restraints	Δρ_min_ = −1.56 e Å^−^^3^

### Special details

**Table:**

Geometry. All e.s.d.'s (except the e.s.d. in the dihedral angle between two l.s. planes) are estimated using the full covariance matrix. The cell e.s.d.'s are taken into account individually in the estimation of e.s.d.'s in distances, angles and torsion angles; correlations between e.s.d.'s in cell parameters are only used when they are defined by crystal symmetry. An approximate (isotropic) treatment of cell e.s.d.'s is used for estimating e.s.d.'s involving l.s. planes.

### Fractional atomic coordinates and isotropic or equivalent isotropic displacement parameters (Å^2^)

**Table:**

	*x*	*y*	*z*	*U*_iso_*/*U*_eq_	Occ. (<1)
Ce1	0.410973 (19)	0.305621 (17)	0.758794 (14)	0.01510 (8)	
Ce2	0.080676 (18)	0.667221 (17)	0.741576 (13)	0.01455 (8)	
S1	0.66051 (10)	0.29972 (9)	0.81865 (7)	0.0236 (2)	
S2	0.46340 (9)	0.22045 (9)	0.58031 (7)	0.0212 (2)	
S3	0.39340 (10)	0.10271 (8)	0.87151 (7)	0.0218 (2)	
S4	0.39297 (8)	0.57690 (8)	0.66960 (6)	0.0173 (2)	
S5	0.23515 (9)	0.47334 (8)	0.91610 (6)	0.01623 (19)	
S6	0.09780 (9)	0.40080 (8)	0.68544 (6)	0.0180 (2)	
S7	0.12669 (9)	0.84940 (8)	0.83149 (7)	0.0210 (2)	
S8	0.02052 (9)	0.77599 (9)	0.55550 (7)	0.0210 (2)	
S9	−0.17573 (9)	0.67948 (9)	0.80323 (7)	0.0231 (2)	
O1	0.5437 (3)	0.3400 (3)	0.8648 (2)	0.0278 (7)	
O2	0.6208 (3)	0.2695 (3)	0.7357 (2)	0.0243 (7)	
O3	0.7347 (3)	0.2085 (3)	0.8696 (3)	0.0372 (8)	
O4	0.7175 (3)	0.3831 (3)	0.7987 (3)	0.0405 (9)	
O5	0.4471 (3)	0.3288 (3)	0.60302 (19)	0.0246 (7)	
O6	0.4531 (3)	0.1610 (3)	0.6682 (2)	0.0241 (6)	
O7	0.3675 (3)	0.2156 (3)	0.5235 (2)	0.0333 (8)	
O8	0.5795 (3)	0.1795 (3)	0.5419 (2)	0.0355 (8)	
O9	0.4956 (3)	0.1498 (3)	0.8581 (2)	0.0312 (8)	
O10	0.2976 (3)	0.1786 (2)	0.8170 (2)	0.0238 (6)	
O11	0.3594 (4)	0.0990 (3)	0.9630 (2)	0.0392 (9)	
O12	0.4245 (3)	−0.0008 (3)	0.8411 (3)	0.0351 (8)	
O13	0.4181 (3)	0.4760 (2)	0.7249 (2)	0.0257 (7)	
O14	0.2622 (3)	0.6211 (3)	0.6645 (2)	0.0250 (7)	
O15	0.4414 (3)	0.5613 (3)	0.5831 (2)	0.0328 (8)	
O16	0.4460 (3)	0.6459 (3)	0.7116 (2)	0.0299 (7)	
O17	0.2921 (3)	0.3836 (3)	0.8656 (2)	0.0306 (8)	
O18	0.1990 (3)	0.5698 (2)	0.85332 (19)	0.0226 (6)	
O19	0.1315 (3)	0.4530 (3)	0.9600 (2)	0.0310 (7)	
O20	0.3203 (4)	0.4870 (3)	0.9764 (2)	0.0389 (9)	
O21	0.2282 (3)	0.3535 (3)	0.6879 (2)	0.0296 (7)	
O22	0.0714 (3)	0.5025 (2)	0.7233 (2)	0.0279 (7)	
O23	0.0569 (3)	0.4180 (3)	0.5965 (2)	0.0415 (9)	
O24	0.0395 (3)	0.3339 (3)	0.7392 (3)	0.0374 (9)	
O25	0.0411 (3)	0.6639 (2)	0.5877 (2)	0.0223 (6)	
O26	0.0327 (3)	0.8243 (2)	0.6372 (2)	0.0244 (7)	
O27	−0.0958 (3)	0.8207 (3)	0.5168 (2)	0.0328 (8)	
O28	0.1162 (3)	0.7889 (3)	0.4947 (2)	0.0324 (8)	
O29	0.2095 (3)	0.7767 (2)	0.7738 (2)	0.0238 (6)	
O30	0.0181 (3)	0.8086 (3)	0.8333 (2)	0.0240 (6)	
O31	0.1753 (3)	0.8365 (3)	0.9184 (2)	0.0344 (8)	
O32	0.0989 (3)	0.9564 (3)	0.7927 (3)	0.0351 (8)	
O33	−0.0600 (3)	0.6309 (3)	0.8508 (2)	0.0243 (7)	
O34	−0.1330 (3)	0.7152 (3)	0.7153 (2)	0.0259 (7)	
O35	−0.2403 (3)	0.6036 (3)	0.7966 (3)	0.0414 (9)	
O36	−0.2466 (3)	0.7696 (3)	0.8444 (2)	0.0373 (9)	
O1W	0.0459 (4)	0.8229 (3)	0.3206 (2)	0.0413 (9)	
H11	0.0422	0.7674	0.3013	0.062*	
H12	0.0761	0.8076	0.3706	0.062*	
O2W	−0.5370 (4)	1.1917 (3)	0.3535 (2)	0.0415 (9)	
H21	−0.5252	1.2493	0.3341	0.062*	
H22	−0.5714	1.1989	0.4021	0.062*	
O3W	0.2480 (6)	1.0066 (4)	0.1103 (4)	0.0817 (18)	
H31	0.3185	0.9835	0.0928	0.122*	
H32	0.2019	1.0111	0.0684	0.122*	
N1	−0.2105 (4)	0.8748 (3)	0.3409 (3)	0.0350 (10)	
H1A	−0.2468	0.8456	0.3078	0.052*	
H1B	−0.2036	0.8419	0.3924	0.052*	
H1C	−0.1408	0.8726	0.3199	0.052*	
N2	−0.2740 (3)	1.1506 (3)	0.3963 (3)	0.0267 (8)	
H2A	−0.2375	1.1795	0.4294	0.040*	
H2B	−0.3476	1.1626	0.4120	0.040*	
H2C	−0.2700	1.1763	0.3430	0.040*	
N3	0.1325 (5)	1.0010 (5)	0.2686 (4)	0.0558 (15)	
H3A	0.0975	0.9533	0.2629	0.084*	
H3B	0.1611	1.0186	0.2191	0.084*	
H3C	0.0819	1.0549	0.2868	0.084*	
N4	0.3721 (4)	1.0569 (4)	0.2712 (3)	0.0430 (11)	
H4A	0.4075	1.1026	0.2821	0.065*	
H4B	0.3295	1.0793	0.2249	0.065*	
H4C	0.4245	0.9989	0.2632	0.065*	
N5	0.5317 (4)	0.5677 (3)	0.8773 (3)	0.0324 (9)	
H5A	0.5075	0.5121	0.8843	0.049*	
H5B	0.6014	0.5545	0.8998	0.049*	
H5C	0.5352	0.5870	0.8224	0.049*	
N6	0.4107 (4)	0.8284 (3)	0.9635 (3)	0.0306 (9)	
H6A	0.4345	0.8841	0.9565	0.046*	
H6B	0.4157	0.8044	1.0177	0.046*	
H6C	0.3378	0.8431	0.9468	0.046*	
N7	0.6676 (4)	0.3958 (4)	0.5790 (3)	0.0357 (10)	
H7A	0.6253	0.4245	0.6212	0.053*	0.50
H7B	0.7083	0.3332	0.5973	0.053*	0.50
H7C	0.6215	0.3934	0.5374	0.053*	0.50
H7'A	0.5985	0.4161	0.6029	0.053*	0.50
H7'B	0.6983	0.3300	0.5939	0.053*	0.50
H7'C	0.6605	0.4067	0.5232	0.053*	0.50
N8	0.8303 (4)	0.6006 (4)	0.5694 (3)	0.0408 (11)	
H8A	0.8190	0.6552	0.5972	0.061*	0.50
H8B	0.8165	0.6198	0.5149	0.061*	0.50
H8C	0.9029	0.5628	0.5762	0.061*	0.50
H8'A	0.7923	0.6337	0.6113	0.061*	0.50
H8'B	0.8394	0.6450	0.5269	0.061*	0.50
H8'C	0.8991	0.5625	0.5877	0.061*	0.50
N9	−0.0783 (4)	0.4128 (3)	0.8926 (3)	0.0276 (8)	
H9A	−0.1431	0.4608	0.9008	0.041*	
H9B	−0.0739	0.3999	0.8389	0.041*	
H9C	−0.0181	0.4338	0.9049	0.041*	
N10	0.1284 (4)	0.2375 (4)	0.9970 (3)	0.0443 (12)	
H10A	0.1916	0.1871	0.9903	0.066*	
H10B	0.1060	0.2342	1.0507	0.066*	
H10C	0.1436	0.2965	0.9827	0.066*	
C1	−0.2796 (5)	0.9847 (4)	0.3455 (4)	0.0319 (11)	
H1D	−0.3588	0.9874	0.3682	0.038*	
H1E	−0.2878	1.0219	0.2875	0.038*	
C2	−0.2175 (4)	1.0364 (4)	0.4038 (3)	0.0280 (10)	
H2D	−0.2218	1.0073	0.4638	0.034*	
H2E	−0.1340	1.0227	0.3873	0.034*	
C3	0.2286 (5)	0.9607 (4)	0.3313 (4)	0.0389 (12)	
H3D	0.2846	0.8993	0.3109	0.047*	
H3E	0.1959	0.9390	0.3865	0.047*	
C4	0.2942 (5)	1.0394 (4)	0.3458 (4)	0.0380 (12)	
H4D	0.2368	1.1055	0.3555	0.046*	
H4E	0.3425	1.0153	0.3979	0.046*	
C5	0.4478 (5)	0.6522 (4)	0.9205 (4)	0.0329 (11)	
H5D	0.3692	0.6667	0.8951	0.039*	
H5E	0.4422	0.6294	0.9819	0.039*	
C6	0.4864 (5)	0.7484 (5)	0.9110 (4)	0.0427 (14)	
H6D	0.4823	0.7762	0.8500	0.051*	
H6E	0.5687	0.7322	0.9296	0.051*	
C7	0.7506 (10)	0.4589 (9)	0.5464 (7)	0.0413 (18)	0.50
H7D	0.8312	0.4132	0.5433	0.050*	0.50
H7E	0.7272	0.4923	0.4882	0.050*	0.50
C8	0.7473 (10)	0.5378 (8)	0.6045 (7)	0.0387 (17)	0.50
H8D	0.7719	0.5047	0.6626	0.046*	0.50
H8E	0.6667	0.5834	0.6081	0.046*	0.50
C7'	0.7458 (10)	0.4569 (9)	0.6093 (7)	0.0413 (18)	0.50
H7'D	0.7105	0.4917	0.6597	0.050*	0.50
H7'E	0.8235	0.4102	0.6257	0.050*	0.50
C8'	0.7581 (10)	0.5332 (8)	0.5388 (7)	0.0387 (17)	0.50
H8'D	0.6799	0.5768	0.5200	0.046*	0.50
H8'E	0.7978	0.4979	0.4897	0.046*	0.50
C9	−0.0779 (4)	0.3166 (4)	0.9493 (4)	0.0358 (11)	
H9D	−0.1463	0.2938	0.9350	0.043*	
H9E	−0.0868	0.3328	1.0095	0.043*	
C10	0.0330 (6)	0.2284 (4)	0.9413 (4)	0.0434 (14)	
H10D	0.0153	0.1621	0.9580	0.052*	
H10E	0.0597	0.2290	0.8811	0.052*	

### Atomic displacement parameters (Å^2^)

**Table:**

	*U*^11^	*U*^22^	*U*^33^	*U*^12^	*U*^13^	*U*^23^
Ce1	0.01515 (12)	0.01357 (12)	0.01596 (12)	−0.00325 (9)	0.00032 (8)	−0.00078 (8)
Ce2	0.01380 (12)	0.01378 (12)	0.01517 (12)	−0.00302 (8)	0.00015 (8)	0.00022 (8)
S1	0.0237 (5)	0.0225 (5)	0.0262 (5)	−0.0094 (4)	−0.0079 (4)	0.0017 (4)
S2	0.0195 (5)	0.0269 (6)	0.0176 (5)	−0.0065 (4)	0.0014 (4)	−0.0044 (4)
S3	0.0240 (5)	0.0179 (5)	0.0226 (5)	−0.0063 (4)	0.0014 (4)	0.0029 (4)
S4	0.0147 (4)	0.0173 (5)	0.0183 (5)	−0.0036 (4)	0.0008 (3)	0.0024 (4)
S5	0.0174 (4)	0.0170 (5)	0.0139 (4)	−0.0042 (4)	−0.0009 (3)	−0.0011 (3)
S6	0.0155 (4)	0.0196 (5)	0.0199 (5)	−0.0051 (4)	−0.0002 (3)	−0.0057 (4)
S7	0.0216 (5)	0.0204 (5)	0.0218 (5)	−0.0064 (4)	−0.0014 (4)	−0.0038 (4)
S8	0.0218 (5)	0.0240 (5)	0.0169 (5)	−0.0073 (4)	−0.0024 (4)	0.0029 (4)
S9	0.0161 (5)	0.0233 (5)	0.0280 (5)	−0.0045 (4)	0.0039 (4)	0.0016 (4)
O1	0.0311 (17)	0.0296 (18)	0.0234 (16)	−0.0077 (14)	−0.0036 (13)	−0.0061 (13)
O2	0.0181 (14)	0.0315 (18)	0.0240 (15)	−0.0070 (13)	−0.0018 (12)	−0.0053 (13)
O3	0.0341 (19)	0.033 (2)	0.043 (2)	−0.0078 (16)	−0.0165 (16)	0.0065 (16)
O4	0.039 (2)	0.036 (2)	0.054 (2)	−0.0247 (17)	−0.0120 (17)	0.0049 (18)
O5	0.0269 (16)	0.0259 (17)	0.0190 (15)	−0.0046 (13)	0.0005 (12)	−0.0015 (12)
O6	0.0290 (16)	0.0242 (16)	0.0205 (15)	−0.0097 (13)	0.0033 (12)	−0.0027 (12)
O7	0.0309 (18)	0.048 (2)	0.0250 (17)	−0.0143 (16)	−0.0048 (13)	−0.0102 (15)
O8	0.0254 (17)	0.051 (2)	0.0260 (17)	−0.0022 (16)	0.0076 (13)	−0.0084 (16)
O9	0.0254 (17)	0.0291 (18)	0.0391 (19)	−0.0116 (14)	−0.0095 (14)	0.0102 (15)
O10	0.0222 (15)	0.0207 (16)	0.0267 (16)	−0.0045 (12)	−0.0007 (12)	0.0012 (12)
O11	0.048 (2)	0.040 (2)	0.0233 (17)	−0.0065 (18)	0.0046 (15)	0.0077 (15)
O12	0.041 (2)	0.0155 (16)	0.047 (2)	−0.0064 (14)	0.0008 (16)	−0.0009 (14)
O13	0.0307 (17)	0.0163 (15)	0.0283 (16)	−0.0059 (13)	−0.0007 (13)	0.0038 (12)
O14	0.0145 (14)	0.0292 (17)	0.0273 (16)	0.0005 (12)	−0.0006 (12)	−0.0026 (13)
O15	0.0289 (17)	0.045 (2)	0.0208 (16)	−0.0055 (15)	0.0075 (13)	0.0005 (14)
O16	0.0326 (18)	0.0262 (18)	0.0357 (18)	−0.0175 (14)	−0.0053 (14)	0.0024 (14)
O17	0.0371 (19)	0.0220 (17)	0.0299 (17)	−0.0036 (14)	0.0121 (14)	−0.0065 (13)
O18	0.0248 (15)	0.0204 (15)	0.0204 (14)	−0.0041 (12)	−0.0030 (12)	0.0027 (12)
O19	0.0264 (16)	0.0330 (19)	0.0332 (18)	−0.0105 (14)	0.0102 (14)	0.0009 (14)
O20	0.049 (2)	0.038 (2)	0.0341 (19)	−0.0196 (18)	−0.0234 (17)	0.0028 (16)
O21	0.0162 (15)	0.0327 (19)	0.0345 (18)	0.0015 (13)	−0.0007 (13)	−0.0019 (14)
O22	0.0339 (18)	0.0183 (16)	0.0316 (17)	−0.0072 (14)	0.0011 (14)	−0.0036 (13)
O23	0.038 (2)	0.058 (3)	0.0269 (18)	−0.0062 (18)	−0.0092 (15)	−0.0131 (17)
O24	0.042 (2)	0.034 (2)	0.045 (2)	−0.0245 (17)	0.0151 (17)	−0.0109 (16)
O25	0.0248 (15)	0.0193 (15)	0.0227 (15)	−0.0067 (12)	−0.0007 (12)	0.0006 (12)
O26	0.0295 (16)	0.0230 (16)	0.0207 (15)	−0.0080 (13)	−0.0017 (12)	−0.0010 (12)
O27	0.0301 (18)	0.0333 (19)	0.0295 (18)	−0.0018 (15)	−0.0106 (14)	0.0058 (14)
O28	0.0364 (19)	0.046 (2)	0.0189 (15)	−0.0208 (17)	0.0047 (13)	0.0018 (14)
O29	0.0234 (15)	0.0243 (16)	0.0234 (15)	−0.0063 (13)	0.0016 (12)	−0.0025 (12)
O30	0.0212 (15)	0.0245 (16)	0.0272 (16)	−0.0071 (13)	0.0024 (12)	−0.0057 (13)
O31	0.0372 (19)	0.047 (2)	0.0231 (17)	−0.0166 (17)	−0.0035 (14)	−0.0087 (15)
O32	0.039 (2)	0.0211 (17)	0.045 (2)	−0.0077 (15)	−0.0047 (16)	0.0010 (15)
O33	0.0194 (14)	0.0246 (16)	0.0257 (16)	−0.0030 (12)	0.0017 (12)	0.0031 (12)
O34	0.0203 (15)	0.0318 (18)	0.0230 (16)	−0.0054 (13)	0.0014 (12)	0.0027 (13)
O35	0.0299 (19)	0.036 (2)	0.062 (3)	−0.0182 (16)	−0.0031 (17)	0.0027 (18)
O36	0.0285 (18)	0.038 (2)	0.038 (2)	0.0031 (15)	0.0065 (15)	−0.0053 (16)
O1W	0.057 (2)	0.038 (2)	0.0344 (19)	−0.0221 (18)	−0.0095 (17)	−0.0007 (16)
O2W	0.053 (2)	0.043 (2)	0.0331 (19)	−0.0232 (19)	0.0055 (17)	−0.0021 (16)
O3W	0.102 (4)	0.049 (3)	0.087 (4)	−0.012 (3)	0.034 (3)	−0.011 (3)
N1	0.036 (2)	0.033 (2)	0.039 (2)	−0.0117 (19)	−0.0032 (18)	−0.0118 (19)
N2	0.0274 (19)	0.023 (2)	0.031 (2)	−0.0109 (16)	0.0077 (16)	−0.0026 (16)
N3	0.038 (3)	0.060 (4)	0.066 (4)	−0.015 (3)	−0.011 (2)	0.014 (3)
N4	0.028 (2)	0.053 (3)	0.047 (3)	−0.012 (2)	0.003 (2)	−0.001 (2)
N5	0.037 (2)	0.026 (2)	0.034 (2)	−0.0062 (18)	−0.0090 (18)	−0.0033 (17)
N6	0.034 (2)	0.028 (2)	0.032 (2)	−0.0128 (18)	0.0010 (17)	−0.0009 (17)
N7	0.039 (2)	0.034 (2)	0.038 (2)	−0.020 (2)	0.0019 (19)	−0.0022 (19)
N8	0.038 (2)	0.042 (3)	0.050 (3)	−0.024 (2)	−0.001 (2)	−0.005 (2)
N9	0.0265 (19)	0.027 (2)	0.028 (2)	−0.0066 (16)	−0.0008 (15)	−0.0035 (16)
N10	0.033 (2)	0.041 (3)	0.053 (3)	−0.002 (2)	−0.005 (2)	0.001 (2)
C1	0.033 (2)	0.024 (2)	0.042 (3)	−0.012 (2)	−0.005 (2)	−0.004 (2)
C2	0.034 (2)	0.022 (2)	0.030 (2)	−0.0106 (19)	−0.0046 (19)	−0.0008 (18)
C3	0.040 (3)	0.028 (3)	0.046 (3)	−0.007 (2)	−0.004 (2)	0.004 (2)
C4	0.041 (3)	0.034 (3)	0.037 (3)	−0.008 (2)	0.007 (2)	−0.002 (2)
C5	0.034 (3)	0.031 (3)	0.036 (3)	−0.012 (2)	0.001 (2)	−0.003 (2)
C6	0.037 (3)	0.037 (3)	0.057 (4)	−0.014 (2)	0.019 (3)	−0.008 (3)
C7	0.043 (4)	0.051 (5)	0.040 (4)	−0.029 (4)	0.007 (4)	−0.007 (4)
C8	0.043 (4)	0.038 (4)	0.041 (4)	−0.024 (3)	0.005 (3)	−0.002 (3)
C7'	0.043 (4)	0.051 (5)	0.040 (4)	−0.029 (4)	0.007 (4)	−0.007 (4)
C8'	0.043 (4)	0.038 (4)	0.041 (4)	−0.024 (3)	0.005 (3)	−0.002 (3)
C9	0.027 (2)	0.035 (3)	0.045 (3)	−0.012 (2)	−0.006 (2)	0.005 (2)
C10	0.061 (4)	0.030 (3)	0.034 (3)	−0.001 (3)	−0.012 (3)	−0.008 (2)

### Geometric parameters (Å, °)

**Table:**

Ce1—O17	2.265 (3)	N3—C3	1.451 (7)
Ce1—O13	2.303 (3)	N3—H3A	0.8600
Ce1—O21	2.323 (3)	N3—H3B	0.8600
Ce1—O2	2.382 (3)	N3—H3C	0.8600
Ce1—O5	2.426 (3)	N4—C4	1.482 (7)
Ce1—O6	2.433 (3)	N4—H4A	0.8600
Ce1—O9	2.434 (3)	N4—H4B	0.8600
Ce1—O1	2.451 (3)	N4—H4C	0.8600
Ce1—O10	2.506 (3)	N5—C5	1.475 (6)
Ce2—O22	2.279 (3)	N5—H5A	0.8600
Ce2—O18	2.290 (3)	N5—H5B	0.8600
Ce2—O14	2.353 (3)	N5—H5C	0.8600
Ce2—O30	2.414 (3)	N6—C6	1.478 (7)
Ce2—O33	2.419 (3)	N6—H6A	0.8600
Ce2—O34	2.432 (3)	N6—H6B	0.8600
Ce2—O25	2.438 (3)	N6—H6C	0.8600
Ce2—O26	2.446 (3)	N7—C7	1.495 (8)
Ce2—O29	2.465 (3)	N7—C7'	1.497 (8)
Ce2—S9	3.0752 (10)	N7—H7A	0.8600
Ce2—S8	3.0830 (10)	N7—H7B	0.8600
Ce2—S7	3.1021 (11)	N7—H7C	0.8600
S1—O3	1.450 (4)	N7—H7'A	0.8599
S1—O4	1.450 (4)	N7—H7'B	0.8599
S1—O1	1.504 (3)	N7—H7'C	0.8600
S1—O2	1.506 (3)	N8—C8	1.502 (8)
S2—O8	1.445 (3)	N8—C8'	1.511 (8)
S2—O7	1.463 (3)	N8—H8A	0.8600
S2—O5	1.483 (3)	N8—H8B	0.8600
S2—O6	1.514 (3)	N8—H8C	0.8600
S3—O11	1.452 (4)	N8—H8'A	0.8600
S3—O12	1.452 (4)	N8—H8'B	0.8603
S3—O10	1.494 (3)	N8—H8'C	0.8598
S3—O9	1.494 (3)	N9—C9	1.473 (7)
S4—O15	1.444 (3)	N9—H9A	0.8600
S4—O16	1.457 (3)	N9—H9B	0.8600
S4—O14	1.477 (3)	N9—H9C	0.8600
S4—O13	1.476 (3)	N10—C10	1.467 (8)
S5—O19	1.444 (3)	N10—H10A	0.8600
S5—O20	1.444 (3)	N10—H10B	0.8600
S5—O17	1.484 (3)	N10—H10C	0.8600
S5—O18	1.494 (3)	C1—C2	1.504 (7)
S6—O23	1.441 (4)	C1—H1D	0.9700
S6—O24	1.447 (4)	C1—H1E	0.9700
S6—O21	1.476 (3)	C2—H2D	0.9700
S6—O22	1.485 (3)	C2—H2E	0.9700
S7—O32	1.440 (4)	C3—C4	1.501 (8)
S7—O31	1.447 (3)	C3—H3D	0.9700
S7—O29	1.507 (3)	C3—H3E	0.9700
S7—O30	1.511 (3)	C4—H4D	0.9700
S8—O27	1.442 (3)	C4—H4E	0.9700
S8—O28	1.469 (3)	C5—C6	1.467 (8)
S8—O25	1.481 (3)	C5—H5D	0.9700
S8—O26	1.502 (3)	C5—H5E	0.9700
S9—O35	1.435 (4)	C6—H6D	0.9700
S9—O36	1.452 (4)	C6—H6E	0.9700
S9—O33	1.503 (3)	C7—C8	1.446 (11)
S9—O34	1.505 (3)	C7—H7D	0.9700
O1W—H11	0.8401	C7—H7E	0.9700
O1W—H12	0.8400	C8—H8D	0.9700
O2W—H21	0.8401	C8—H8E	0.9700
O2W—H22	0.8400	C7'—C8'	1.440 (11)
O3W—H31	0.8401	C7'—H7'D	0.9700
O3W—H32	0.8401	C7'—H7'E	0.9700
N1—C1	1.471 (6)	C8'—H8'D	0.9700
N1—H1A	0.8600	C8'—H8'E	0.9700
N1—H1B	0.8600	C9—C10	1.499 (8)
N1—H1C	0.8600	C9—H9D	0.9700
N2—C2	1.477 (6)	C9—H9E	0.9700
N2—H2A	0.8600	C10—H10D	0.9700
N2—H2B	0.8600	C10—H10E	0.9700
N2—H2C	0.8600		
			
O17—Ce1—O13	80.01 (12)	O34—S9—Ce2	51.12 (12)
O17—Ce1—O21	80.34 (13)	S1—O1—Ce1	98.64 (15)
O13—Ce1—O21	87.44 (12)	S1—O2—Ce1	101.51 (15)
O17—Ce1—O2	131.34 (12)	S2—O5—Ce1	100.54 (16)
O13—Ce1—O2	82.31 (11)	S2—O6—Ce1	99.30 (16)
O21—Ce1—O2	143.51 (11)	S3—O9—Ce1	102.10 (17)
O17—Ce1—O5	141.40 (12)	S3—O10—Ce1	98.97 (15)
O13—Ce1—O5	72.80 (11)	S4—O13—Ce1	153.8 (2)
O21—Ce1—O5	71.73 (11)	S4—O14—Ce2	146.8 (2)
O2—Ce1—O5	71.79 (11)	S5—O17—Ce1	153.9 (2)
O17—Ce1—O6	144.82 (12)	S5—O18—Ce2	150.4 (2)
O13—Ce1—O6	129.96 (11)	S6—O21—Ce1	151.9 (2)
O21—Ce1—O6	82.96 (11)	S6—O22—Ce2	158.4 (2)
O2—Ce1—O6	77.31 (11)	S8—O25—Ce2	100.89 (16)
O5—Ce1—O6	57.53 (11)	S8—O26—Ce2	99.91 (16)
O17—Ce1—O9	91.38 (13)	S7—O29—Ce2	99.91 (15)
O13—Ce1—O9	141.67 (12)	S7—O30—Ce2	101.98 (15)
O21—Ce1—O9	128.13 (12)	S9—O33—Ce2	100.69 (15)
O2—Ce1—O9	75.69 (11)	S9—O34—Ce2	100.08 (16)
O5—Ce1—O9	126.81 (12)	H11—O1W—H12	108.4
O6—Ce1—O9	75.13 (12)	H21—O2W—H22	108.4
O17—Ce1—O1	73.66 (12)	H31—O3W—H32	108.2
O13—Ce1—O1	72.84 (12)	C1—N1—H1A	109.5
O21—Ce1—O1	149.54 (11)	C1—N1—H1B	109.5
O2—Ce1—O1	57.83 (11)	H1A—N1—H1B	109.5
O5—Ce1—O1	121.46 (11)	C1—N1—H1C	109.5
O6—Ce1—O1	127.49 (11)	H1A—N1—H1C	109.5
O9—Ce1—O1	68.91 (12)	H1B—N1—H1C	109.5
O17—Ce1—O10	74.58 (11)	C2—N2—H2A	109.5
O13—Ce1—O10	149.60 (11)	C2—N2—H2B	109.5
O21—Ce1—O10	72.23 (11)	H2A—N2—H2B	109.5
O2—Ce1—O10	127.28 (11)	C2—N2—H2C	109.5
O5—Ce1—O10	119.21 (11)	H2A—N2—H2C	109.5
O6—Ce1—O10	70.90 (10)	H2B—N2—H2C	109.5
O9—Ce1—O10	56.32 (10)	C3—N3—H3A	109.5
O1—Ce1—O10	114.49 (11)	C3—N3—H3B	109.5
O22—Ce2—O18	78.58 (12)	H3A—N3—H3B	109.5
O22—Ce2—O14	85.12 (12)	C3—N3—H3C	109.5
O18—Ce2—O14	81.55 (11)	H3A—N3—H3C	109.5
O22—Ce2—O30	144.24 (11)	H3B—N3—H3C	109.5
O18—Ce2—O30	88.54 (11)	C4—N4—H4A	109.5
O14—Ce2—O30	126.13 (11)	C4—N4—H4B	109.5
O22—Ce2—O33	75.05 (11)	H4A—N4—H4B	109.5
O18—Ce2—O33	76.07 (11)	C4—N4—H4C	109.5
O14—Ce2—O33	152.51 (11)	H4A—N4—H4C	109.5
O30—Ce2—O33	69.48 (11)	H4B—N4—H4C	109.5
O22—Ce2—O34	83.91 (12)	C5—N5—H5A	109.5
O18—Ce2—O34	133.17 (11)	C5—N5—H5B	109.5
O14—Ce2—O34	139.84 (11)	H5A—N5—H5B	109.5
O30—Ce2—O34	80.98 (11)	C5—N5—H5C	109.5
O33—Ce2—O34	57.46 (10)	H5A—N5—H5C	109.5
O22—Ce2—O25	72.50 (11)	H5B—N5—H5C	109.5
O18—Ce2—O25	140.83 (10)	C6—N6—H6A	109.5
O14—Ce2—O25	70.33 (11)	C6—N6—H6B	109.5
O30—Ce2—O25	130.01 (11)	H6A—N6—H6B	109.5
O33—Ce2—O25	119.58 (11)	C6—N6—H6C	109.5
O34—Ce2—O25	69.52 (10)	H6A—N6—H6C	109.5
O22—Ce2—O26	128.77 (11)	H6B—N6—H6C	109.5
O18—Ce2—O26	148.16 (11)	C7—N7—H7A	109.5
O14—Ce2—O26	84.82 (11)	C7—N7—H7B	109.5
O30—Ce2—O26	76.56 (11)	H7A—N7—H7B	109.5
O33—Ce2—O26	122.45 (10)	C7—N7—H7C	109.5
O34—Ce2—O26	72.66 (11)	H7A—N7—H7C	109.5
O25—Ce2—O26	56.82 (10)	H7B—N7—H7C	109.5
O22—Ce2—O29	146.07 (11)	C7'—N7—H7'A	108.9
O18—Ce2—O29	76.10 (11)	C7'—N7—H7'B	109.8
O14—Ce2—O29	69.20 (11)	H7A—N7—H7'B	109.9
O30—Ce2—O29	57.04 (10)	H7'A—N7—H7'B	109.5
O33—Ce2—O29	119.33 (11)	C7'—N7—H7'C	109.8
O34—Ce2—O29	130.00 (11)	H7'A—N7—H7'C	109.5
O25—Ce2—O29	116.08 (10)	H7'B—N7—H7'C	109.5
O26—Ce2—O29	72.17 (11)	C8—N8—H8A	109.5
O22—Ce2—S9	79.08 (9)	C8—N8—H8B	109.5
O18—Ce2—S9	104.70 (8)	H8A—N8—H8B	109.5
O14—Ce2—S9	161.34 (8)	C8—N8—H8C	109.5
O30—Ce2—S9	72.12 (8)	H8A—N8—H8C	109.5
O33—Ce2—S9	28.70 (8)	H8B—N8—H8C	109.5
O34—Ce2—S9	28.80 (8)	C8'—N8—H8'A	108.7
O25—Ce2—S9	95.26 (8)	C8'—N8—H8'B	109.3
O26—Ce2—S9	97.34 (8)	H8'A—N8—H8'B	109.4
O29—Ce2—S9	129.16 (7)	H8'A—N8—H8'C	109.5
O22—Ce2—S8	100.48 (9)	H8'B—N8—H8'C	109.5
O18—Ce2—S8	156.87 (8)	C9—N9—H9A	109.5
O14—Ce2—S8	75.35 (8)	C9—N9—H9B	109.5
O30—Ce2—S8	103.93 (8)	H9A—N9—H9B	109.5
O33—Ce2—S8	126.39 (8)	C9—N9—H9C	109.5
O34—Ce2—S8	68.93 (8)	H9A—N9—H9C	109.5
O25—Ce2—S8	28.15 (7)	H9B—N9—H9C	109.5
O26—Ce2—S8	28.68 (7)	C10—N10—H10A	109.5
O29—Ce2—S8	94.21 (8)	C10—N10—H10B	109.5
S9—Ce2—S8	97.71 (3)	H10A—N10—H10B	109.5
O22—Ce2—S7	159.18 (9)	C10—N10—H10C	109.5
O18—Ce2—S7	81.44 (8)	H10A—N10—H10C	109.5
O14—Ce2—S7	97.72 (8)	H10B—N10—H10C	109.5
O30—Ce2—S7	28.46 (8)	N1—C1—C2	110.0 (4)
O33—Ce2—S7	94.55 (8)	N1—C1—H1D	109.7
O34—Ce2—S7	105.95 (8)	C2—C1—H1D	109.7
O25—Ce2—S7	127.93 (8)	N1—C1—H1E	109.7
O26—Ce2—S7	72.04 (8)	C2—C1—H1E	109.7
O29—Ce2—S7	28.58 (7)	H1D—C1—H1E	108.2
S9—Ce2—S7	100.57 (3)	N2—C2—C1	110.2 (4)
S8—Ce2—S7	100.19 (3)	N2—C2—H2D	109.6
O3—S1—O4	113.5 (2)	C1—C2—H2D	109.6
O3—S1—O1	110.0 (2)	N2—C2—H2E	109.6
O4—S1—O1	110.2 (2)	C1—C2—H2E	109.6
O3—S1—O2	110.7 (2)	H2D—C2—H2E	108.1
O4—S1—O2	110.0 (2)	N3—C3—C4	113.0 (5)
O1—S1—O2	101.92 (18)	N3—C3—H3D	109.0
O8—S2—O7	111.6 (2)	C4—C3—H3D	109.0
O8—S2—O5	111.1 (2)	N3—C3—H3E	109.0
O7—S2—O5	111.1 (2)	C4—C3—H3E	109.0
O8—S2—O6	110.8 (2)	H3D—C3—H3E	107.8
O7—S2—O6	109.3 (2)	N4—C4—C3	112.6 (5)
O5—S2—O6	102.56 (18)	N4—C4—H4D	109.1
O11—S3—O12	111.4 (2)	C3—C4—H4D	109.1
O11—S3—O10	110.9 (2)	N4—C4—H4E	109.1
O12—S3—O10	111.6 (2)	C3—C4—H4E	109.1
O11—S3—O9	109.6 (2)	H4D—C4—H4E	107.8
O12—S3—O9	110.5 (2)	C6—C5—N5	111.4 (4)
O10—S3—O9	102.60 (19)	C6—C5—H5D	109.3
O15—S4—O16	111.1 (2)	N5—C5—H5D	109.3
O15—S4—O14	109.6 (2)	C6—C5—H5E	109.3
O16—S4—O14	110.2 (2)	N5—C5—H5E	109.3
O15—S4—O13	110.5 (2)	H5D—C5—H5E	108.0
O16—S4—O13	107.6 (2)	C5—C6—N6	111.1 (4)
O14—S4—O13	107.80 (19)	C5—C6—H6D	109.4
O19—S5—O20	112.2 (2)	N6—C6—H6D	109.4
O19—S5—O17	108.2 (2)	C5—C6—H6E	109.4
O20—S5—O17	109.7 (2)	N6—C6—H6E	109.4
O19—S5—O18	110.22 (19)	H6D—C6—H6E	108.0
O20—S5—O18	108.5 (2)	C8—C7—N7	109.7 (7)
O17—S5—O18	107.99 (19)	C8—C7—H7D	109.7
O23—S6—O24	111.7 (2)	N7—C7—H7D	109.7
O23—S6—O21	109.8 (2)	H7'C—C7—H7D	111.8
O24—S6—O21	109.7 (2)	C8—C7—H7E	109.7
O23—S6—O22	109.7 (2)	N7—C7—H7E	109.7
O24—S6—O22	107.9 (2)	H7D—C7—H7E	108.2
O21—S6—O22	108.0 (2)	C7—C8—N8	108.4 (7)
O32—S7—O31	112.6 (2)	C7—C8—H8D	110.0
O32—S7—O29	111.0 (2)	N8—C8—H8D	110.0
O31—S7—O29	110.8 (2)	C7—C8—H8E	110.0
O32—S7—O30	110.9 (2)	N8—C8—H8E	110.0
O31—S7—O30	110.0 (2)	H8D—C8—H8E	108.4
O29—S7—O30	101.07 (18)	C8'—C7'—N7	108.2 (7)
O32—S7—Ce2	123.95 (16)	C8'—C7'—H7'D	110.1
O31—S7—Ce2	123.43 (17)	N7—C7'—H7'D	110.1
O29—S7—Ce2	51.51 (13)	C8'—C7'—H7'E	110.1
O30—S7—Ce2	49.56 (12)	N7—C7'—H7'E	110.1
O27—S8—O28	111.7 (2)	H7'D—C7'—H7'E	108.4
O27—S8—O25	112.3 (2)	C7'—C8'—N8	109.3 (7)
O28—S8—O25	109.9 (2)	C7'—C8'—H8'D	109.8
O27—S8—O26	111.7 (2)	N8—C8'—H8'D	109.8
O28—S8—O26	108.5 (2)	C7'—C8'—H8'E	109.8
O25—S8—O26	102.34 (18)	N8—C8'—H8'E	109.8
O27—S8—Ce2	127.91 (15)	H8'D—C8'—H8'E	108.3
O28—S8—Ce2	120.40 (15)	N9—C9—C10	113.8 (5)
O25—S8—Ce2	50.96 (12)	N9—C9—H9D	108.8
O26—S8—Ce2	51.41 (13)	C10—C9—H9D	108.8
O35—S9—O36	111.8 (2)	N9—C9—H9E	108.8
O35—S9—O33	111.2 (2)	C10—C9—H9E	108.8
O36—S9—O33	110.7 (2)	H9D—C9—H9E	107.7
O35—S9—O34	111.4 (2)	N10—C10—C9	111.2 (5)
O36—S9—O34	109.7 (2)	N10—C10—H10D	109.4
O33—S9—O34	101.66 (18)	C9—C10—H10D	109.4
O35—S9—Ce2	127.53 (17)	N10—C10—H10E	109.4
O36—S9—Ce2	120.65 (17)	C9—C10—H10E	109.4
O33—S9—Ce2	50.62 (12)	H10D—C10—H10E	108.0
			
O22—Ce2—S7—O32	−176.1 (3)	O2—Ce1—O9—S3	−156.8 (2)
O18—Ce2—S7—O32	167.5 (2)	O5—Ce1—O9—S3	−103.2 (2)
O14—Ce2—S7—O32	87.3 (2)	O6—Ce1—O9—S3	−76.37 (19)
O30—Ce2—S7—O32	−89.5 (3)	O1—Ce1—O9—S3	142.6 (2)
O33—Ce2—S7—O32	−117.3 (2)	O10—Ce1—O9—S3	0.33 (15)
O34—Ce2—S7—O32	−59.9 (2)	O11—S3—O10—Ce1	117.4 (2)
O25—Ce2—S7—O32	16.3 (2)	O12—S3—O10—Ce1	−117.80 (19)
O26—Ce2—S7—O32	5.4 (2)	O9—S3—O10—Ce1	0.5 (2)
O29—Ce2—S7—O32	90.9 (2)	O17—Ce1—O10—S3	−102.68 (17)
S9—Ce2—S7—O32	−88.99 (19)	O13—Ce1—O10—S3	−136.97 (19)
S8—Ce2—S7—O32	10.9 (2)	O21—Ce1—O10—S3	172.74 (18)
O22—Ce2—S7—O31	2.0 (3)	O2—Ce1—O10—S3	28.0 (2)
O18—Ce2—S7—O31	−14.4 (2)	O5—Ce1—O10—S3	116.56 (15)
O14—Ce2—S7—O31	−94.6 (2)	O6—Ce1—O10—S3	84.19 (16)
O30—Ce2—S7—O31	88.5 (2)	O9—Ce1—O10—S3	−0.33 (15)
O33—Ce2—S7—O31	60.8 (2)	O1—Ce1—O10—S3	−39.19 (18)
O34—Ce2—S7—O31	118.2 (2)	O15—S4—O13—Ce1	−57.2 (5)
O25—Ce2—S7—O31	−165.6 (2)	O16—S4—O13—Ce1	−178.6 (4)
O26—Ce2—S7—O31	−176.5 (2)	O14—S4—O13—Ce1	62.6 (5)
O29—Ce2—S7—O31	−91.0 (2)	O17—Ce1—O13—S4	−112.6 (5)
S9—Ce2—S7—O31	89.08 (18)	O21—Ce1—O13—S4	−32.0 (5)
S8—Ce2—S7—O31	−170.99 (18)	O2—Ce1—O13—S4	112.9 (5)
O22—Ce2—S7—O29	93.0 (3)	O5—Ce1—O13—S4	39.7 (4)
O18—Ce2—S7—O29	76.62 (18)	O6—Ce1—O13—S4	46.6 (5)
O14—Ce2—S7—O29	−3.57 (17)	O9—Ce1—O13—S4	168.0 (4)
O30—Ce2—S7—O29	179.6 (2)	O1—Ce1—O13—S4	171.6 (5)
O33—Ce2—S7—O29	151.77 (17)	O10—Ce1—O13—S4	−79.1 (5)
O34—Ce2—S7—O29	−150.81 (17)	O15—S4—O14—Ce2	171.4 (3)
O25—Ce2—S7—O29	−74.62 (18)	O16—S4—O14—Ce2	−66.0 (4)
O26—Ce2—S7—O29	−85.54 (18)	O13—S4—O14—Ce2	51.1 (4)
S9—Ce2—S7—O29	−179.91 (16)	O22—Ce2—O14—S4	−92.0 (4)
S8—Ce2—S7—O29	−79.99 (16)	O18—Ce2—O14—S4	−12.8 (4)
O22—Ce2—S7—O30	−86.5 (3)	O30—Ce2—O14—S4	69.1 (4)
O18—Ce2—S7—O30	−102.94 (19)	O33—Ce2—O14—S4	−48.5 (5)
O14—Ce2—S7—O30	176.87 (19)	O34—Ce2—O14—S4	−166.6 (3)
O33—Ce2—S7—O30	−27.79 (18)	O25—Ce2—O14—S4	−165.1 (4)
O34—Ce2—S7—O30	29.64 (19)	O26—Ce2—O14—S4	138.3 (4)
O25—Ce2—S7—O30	105.83 (19)	O29—Ce2—O14—S4	65.4 (4)
O26—Ce2—S7—O30	94.90 (19)	S9—Ce2—O14—S4	−124.1 (3)
O29—Ce2—S7—O30	−179.6 (2)	S8—Ce2—O14—S4	165.8 (4)
S9—Ce2—S7—O30	0.54 (17)	S7—Ce2—O14—S4	67.2 (4)
S8—Ce2—S7—O30	100.46 (17)	O19—S5—O17—Ce1	159.2 (5)
O22—Ce2—S8—O27	82.8 (2)	O20—S5—O17—Ce1	−78.1 (6)
O18—Ce2—S8—O27	168.2 (3)	O18—S5—O17—Ce1	39.9 (6)
O14—Ce2—S8—O27	164.9 (2)	O13—Ce1—O17—S5	−6.2 (5)
O30—Ce2—S8—O27	−70.9 (2)	O21—Ce1—O17—S5	−95.3 (5)
O33—Ce2—S8—O27	3.7 (2)	O2—Ce1—O17—S5	64.2 (6)
O34—Ce2—S8—O27	3.5 (2)	O5—Ce1—O17—S5	−51.6 (6)
O25—Ce2—S8—O27	89.3 (3)	O6—Ce1—O17—S5	−158.1 (4)
O26—Ce2—S8—O27	−88.7 (3)	O9—Ce1—O17—S5	136.2 (5)
O29—Ce2—S8—O27	−127.9 (2)	O1—Ce1—O17—S5	68.7 (5)
S9—Ce2—S8—O27	2.5 (2)	O10—Ce1—O17—S5	−169.4 (6)
S7—Ce2—S8—O27	−99.7 (2)	O19—S5—O18—Ce2	−49.6 (4)
O22—Ce2—S8—O28	−98.3 (2)	O20—S5—O18—Ce2	−172.9 (4)
O18—Ce2—S8—O28	−12.9 (3)	O17—S5—O18—Ce2	68.3 (4)
O14—Ce2—S8—O28	−16.3 (2)	O22—Ce2—O18—S5	−23.5 (4)
O30—Ce2—S8—O28	108.0 (2)	O14—Ce2—O18—S5	−110.2 (4)
O33—Ce2—S8—O28	−177.4 (2)	O30—Ce2—O18—S5	122.9 (4)
O34—Ce2—S8—O28	−177.6 (2)	O33—Ce2—O18—S5	53.7 (4)
O25—Ce2—S8—O28	−91.9 (2)	O34—Ce2—O18—S5	46.7 (4)
O26—Ce2—S8—O28	90.2 (2)	O25—Ce2—O18—S5	−66.3 (4)
O29—Ce2—S8—O28	50.9 (2)	O26—Ce2—O18—S5	−175.9 (3)
S9—Ce2—S8—O28	−178.60 (18)	O29—Ce2—O18—S5	179.2 (4)
S7—Ce2—S8—O28	79.13 (18)	S9—Ce2—O18—S5	51.8 (4)
O22—Ce2—S8—O25	−6.48 (18)	S8—Ce2—O18—S5	−113.5 (4)
O18—Ce2—S8—O25	79.0 (3)	S7—Ce2—O18—S5	150.6 (4)
O14—Ce2—S8—O25	75.60 (18)	O23—S6—O21—Ce1	165.8 (5)
O30—Ce2—S8—O25	−160.13 (17)	O24—S6—O21—Ce1	−71.0 (5)
O33—Ce2—S8—O25	−85.55 (18)	O22—S6—O21—Ce1	46.3 (5)
O34—Ce2—S8—O25	−85.71 (18)	O17—Ce1—O21—S6	−1.6 (5)
O26—Ce2—S8—O25	−177.9 (2)	O13—Ce1—O21—S6	−81.9 (5)
O29—Ce2—S8—O25	142.81 (17)	O2—Ce1—O21—S6	−155.3 (4)
S9—Ce2—S8—O25	−86.73 (16)	O5—Ce1—O21—S6	−154.6 (5)
S7—Ce2—S8—O25	171.00 (16)	O6—Ce1—O21—S6	147.3 (5)
O22—Ce2—S8—O26	171.46 (18)	O9—Ce1—O21—S6	82.5 (5)
O18—Ce2—S8—O26	−103.1 (3)	O1—Ce1—O21—S6	−33.1 (6)
O14—Ce2—S8—O26	−106.46 (18)	O10—Ce1—O21—S6	75.2 (5)
O30—Ce2—S8—O26	17.81 (18)	O23—S6—O22—Ce2	−64.6 (6)
O33—Ce2—S8—O26	92.39 (19)	O24—S6—O22—Ce2	173.5 (6)
O34—Ce2—S8—O26	92.23 (18)	O21—S6—O22—Ce2	55.1 (6)
O25—Ce2—S8—O26	177.9 (2)	O18—Ce2—O22—S6	−101.9 (6)
O29—Ce2—S8—O26	−39.25 (18)	O14—Ce2—O22—S6	−19.6 (6)
S9—Ce2—S8—O26	91.21 (16)	O30—Ce2—O22—S6	−173.0 (5)
S7—Ce2—S8—O26	−11.06 (16)	O33—Ce2—O22—S6	179.6 (6)
O22—Ce2—S9—O35	−8.8 (2)	O34—Ce2—O22—S6	121.7 (6)
O18—Ce2—S9—O35	−83.8 (2)	O25—Ce2—O22—S6	51.3 (6)
O14—Ce2—S9—O35	23.8 (4)	O26—Ce2—O22—S6	59.8 (6)
O30—Ce2—S9—O35	−167.4 (2)	O29—Ce2—O22—S6	−59.6 (7)
O33—Ce2—S9—O35	−87.6 (3)	S9—Ce2—O22—S6	150.4 (6)
O34—Ce2—S9—O35	88.4 (3)	S8—Ce2—O22—S6	54.5 (6)
O25—Ce2—S9—O35	62.2 (2)	S7—Ce2—O22—S6	−118.5 (5)
O26—Ce2—S9—O35	119.4 (2)	O27—S8—O25—Ce2	−121.52 (18)
O29—Ce2—S9—O35	−167.7 (2)	O28—S8—O25—Ce2	113.52 (18)
S8—Ce2—S9—O35	90.4 (2)	O26—S8—O25—Ce2	−1.65 (18)
S7—Ce2—S9—O35	−167.6 (2)	O22—Ce2—O25—S8	173.32 (18)
O22—Ce2—S9—O36	171.3 (2)	O18—Ce2—O25—S8	−142.37 (16)
O18—Ce2—S9—O36	96.3 (2)	O14—Ce2—O25—S8	−95.66 (17)
O14—Ce2—S9—O36	−156.1 (3)	O30—Ce2—O25—S8	25.5 (2)
O30—Ce2—S9—O36	12.8 (2)	O33—Ce2—O25—S8	112.65 (16)
O33—Ce2—S9—O36	92.5 (3)	O34—Ce2—O25—S8	83.37 (16)
O34—Ce2—S9—O36	−91.4 (3)	O26—Ce2—O25—S8	1.18 (13)
O25—Ce2—S9—O36	−117.7 (2)	O29—Ce2—O25—S8	−42.16 (18)
O26—Ce2—S9—O36	−60.5 (2)	S9—Ce2—O25—S8	96.52 (14)
O29—Ce2—S9—O36	12.4 (2)	S7—Ce2—O25—S8	−11.26 (19)
S8—Ce2—S9—O36	−89.45 (19)	O27—S8—O26—Ce2	121.93 (19)
S7—Ce2—S9—O36	12.49 (19)	O28—S8—O26—Ce2	−114.53 (19)
O22—Ce2—S9—O33	78.77 (19)	O25—S8—O26—Ce2	1.64 (18)
O18—Ce2—S9—O33	3.79 (19)	O22—Ce2—O26—S8	−10.8 (2)
O14—Ce2—S9—O33	111.4 (3)	O18—Ce2—O26—S8	133.51 (18)
O30—Ce2—S9—O33	−79.77 (19)	O14—Ce2—O26—S8	68.69 (16)
O34—Ce2—S9—O33	176.1 (2)	O30—Ce2—O26—S8	−162.23 (18)
O25—Ce2—S9—O33	149.79 (18)	O33—Ce2—O26—S8	−107.61 (17)
O26—Ce2—S9—O33	−153.04 (18)	O34—Ce2—O26—S8	−77.64 (16)
O29—Ce2—S9—O33	−80.09 (19)	O25—Ce2—O26—S8	−1.16 (13)
S8—Ce2—S9—O33	178.02 (17)	O29—Ce2—O26—S8	138.48 (18)
S7—Ce2—S9—O33	−80.03 (17)	S9—Ce2—O26—S8	−92.66 (15)
O22—Ce2—S9—O34	−97.3 (2)	S7—Ce2—O26—S8	168.55 (17)
O18—Ce2—S9—O34	−172.26 (19)	O32—S7—O29—Ce2	−117.34 (19)
O14—Ce2—S9—O34	−64.7 (3)	O31—S7—O29—Ce2	116.83 (19)
O30—Ce2—S9—O34	104.18 (19)	O30—S7—O29—Ce2	0.34 (18)
O33—Ce2—S9—O34	−176.1 (2)	O22—Ce2—O29—S7	−140.51 (18)
O25—Ce2—S9—O34	−26.26 (19)	O18—Ce2—O29—S7	−97.68 (16)
O26—Ce2—S9—O34	30.91 (19)	O14—Ce2—O29—S7	176.21 (18)
O29—Ce2—S9—O34	103.9 (2)	O30—Ce2—O29—S7	−0.25 (13)
S8—Ce2—S9—O34	1.97 (18)	O33—Ce2—O29—S7	−32.74 (19)
S7—Ce2—S9—O34	103.92 (18)	O34—Ce2—O29—S7	37.8 (2)
O3—S1—O1—Ce1	−114.73 (19)	O25—Ce2—O29—S7	122.14 (15)
O4—S1—O1—Ce1	119.4 (2)	O26—Ce2—O29—S7	85.02 (16)
O2—S1—O1—Ce1	2.73 (19)	S9—Ce2—O29—S7	0.1 (2)
O17—Ce1—O1—S1	−178.0 (2)	S8—Ce2—O29—S7	103.63 (14)
O13—Ce1—O1—S1	−93.65 (18)	O32—S7—O30—Ce2	117.4 (2)
O21—Ce1—O1—S1	−145.53 (19)	O31—S7—O30—Ce2	−117.43 (19)
O2—Ce1—O1—S1	−2.00 (14)	O29—S7—O30—Ce2	−0.35 (19)
O5—Ce1—O1—S1	−37.1 (2)	O22—Ce2—O30—S7	142.62 (18)
O6—Ce1—O1—S1	34.0 (2)	O18—Ce2—O30—S7	74.59 (17)
O9—Ce1—O1—S1	83.97 (18)	O14—Ce2—O30—S7	−3.8 (2)
O10—Ce1—O1—S1	117.99 (16)	O33—Ce2—O30—S7	150.25 (19)
O3—S1—O2—Ce1	114.1 (2)	O34—Ce2—O30—S7	−151.22 (18)
O4—S1—O2—Ce1	−119.7 (2)	O25—Ce2—O30—S7	−97.79 (18)
O1—S1—O2—Ce1	−2.8 (2)	O26—Ce2—O30—S7	−77.02 (17)
O17—Ce1—O2—S1	7.1 (3)	O29—Ce2—O30—S7	0.25 (13)
O13—Ce1—O2—S1	76.53 (17)	S9—Ce2—O30—S7	−179.45 (18)
O21—Ce1—O2—S1	151.57 (17)	S8—Ce2—O30—S7	−85.70 (15)
O5—Ce1—O2—S1	150.9 (2)	O35—S9—O33—Ce2	121.8 (2)
O6—Ce1—O2—S1	−149.47 (19)	O36—S9—O33—Ce2	−113.3 (2)
O9—Ce1—O2—S1	−71.83 (18)	O34—S9—O33—Ce2	3.1 (2)
O1—Ce1—O2—S1	2.01 (14)	O22—Ce2—O33—S9	−94.56 (18)
O10—Ce1—O2—S1	−95.84 (18)	O18—Ce2—O33—S9	−176.22 (19)
O8—S2—O5—Ce1	−120.89 (18)	O14—Ce2—O33—S9	−139.8 (2)
O7—S2—O5—Ce1	114.21 (18)	O30—Ce2—O33—S9	90.05 (17)
O6—S2—O5—Ce1	−2.42 (19)	O34—Ce2—O33—S9	−2.26 (14)
O17—Ce1—O5—S2	−137.35 (18)	O25—Ce2—O33—S9	−35.2 (2)
O13—Ce1—O5—S2	175.42 (18)	O26—Ce2—O33—S9	32.2 (2)
O21—Ce1—O5—S2	−91.54 (18)	O29—Ce2—O33—S9	118.82 (16)
O2—Ce1—O5—S2	88.03 (17)	S8—Ce2—O33—S9	−2.4 (2)
O6—Ce1—O5—S2	1.74 (13)	S7—Ce2—O33—S9	103.77 (15)
O9—Ce1—O5—S2	32.9 (2)	O35—S9—O34—Ce2	−121.6 (2)
O1—Ce1—O5—S2	118.90 (16)	O36—S9—O34—Ce2	114.0 (2)
O10—Ce1—O5—S2	−35.11 (19)	O33—S9—O34—Ce2	−3.1 (2)
O8—S2—O6—Ce1	121.1 (2)	O22—Ce2—O34—S9	78.38 (18)
O7—S2—O6—Ce1	−115.55 (18)	O18—Ce2—O34—S9	10.3 (3)
O5—S2—O6—Ce1	2.40 (18)	O14—Ce2—O34—S9	153.36 (15)
O17—Ce1—O6—S2	133.14 (19)	O30—Ce2—O34—S9	−69.10 (17)
O13—Ce1—O6—S2	−9.6 (2)	O33—Ce2—O34—S9	2.25 (14)
O21—Ce1—O6—S2	71.09 (16)	O25—Ce2—O34—S9	152.0 (2)
O2—Ce1—O6—S2	−78.02 (16)	O26—Ce2—O34—S9	−147.74 (19)
O5—Ce1—O6—S2	−1.70 (13)	O29—Ce2—O34—S9	−100.64 (18)
O9—Ce1—O6—S2	−156.35 (18)	S8—Ce2—O34—S9	−177.90 (19)
O1—Ce1—O6—S2	−108.65 (17)	S7—Ce2—O34—S9	−82.90 (16)
O10—Ce1—O6—S2	144.67 (18)	N1—C1—C2—N2	169.9 (4)
O11—S3—O9—Ce1	−118.3 (2)	N3—C3—C4—N4	−73.2 (6)
O12—S3—O9—Ce1	118.6 (2)	N5—C5—C6—N6	−172.9 (4)
O10—S3—O9—Ce1	−0.5 (2)	N7—C7—C8—N8	−179.3 (8)
O17—Ce1—O9—S3	70.7 (2)	N7—C7'—C8'—N8	176.4 (8)
O13—Ce1—O9—S3	146.27 (17)	N9—C9—C10—N10	84.2 (6)
O21—Ce1—O9—S3	−8.1 (3)		

### Hydrogen-bond geometry (Å, °)

**Table:**

*D*—H···*A*	*D*—H	H···*A*	*D*···*A*	*D*—H···*A*
O1w—H11···O24^i^	0.84	2.02	2.803 (5)	156
O1w—H12···O28	0.84	1.96	2.786 (5)	169
O2w—H21···O16^ii^	0.84	1.94	2.754 (5)	162
O2w—H22···O7^iii^	0.84	1.99	2.828 (5)	173
O3w—H31···O9^iv^	0.84	2.47	3.153 (7)	139
N1—H1a···O10^i^	0.86	2.11	2.899 (5)	151
N1—H1b···O27	0.86	2.28	2.991 (6)	141
N1—H1c···O1w	0.86	2.09	2.891 (6)	154
N2—H2a···O28^ii^	0.86	2.02	2.863 (5)	165
N2—H2b···O8^iii^	0.86	2.16	2.769 (5)	128
N2—H2c···O29^ii^	0.86	2.01	2.849 (5)	163
N3—H3a···O1w	0.86	2.10	2.863 (7)	148
N3—H3b···O3w	0.86	1.93	2.753 (8)	160
N3—H3c···O26^ii^	0.86	2.20	3.048 (7)	167
N4—H4a···O2w^v^	0.86	1.95	2.760 (7)	157
N4—H4b···O36^ii^	0.86	2.16	2.843 (6)	136
N4—H4c···O6^iv^	0.86	2.37	3.119 (6)	145
N5—H5a···O1	0.86	2.27	3.029 (5)	148
N5—H5b···O20^vi^	0.86	2.09	2.794 (5)	138
N5—H5c···O16	0.86	2.00	2.763 (5)	147
N6—H6a···O12^vii^	0.86	2.21	2.837 (6)	130
N6—H6b···O1^vi^	0.86	2.46	3.241 (5)	152
N6—H6c···O31	0.86	1.99	2.826 (6)	165
N7—H7'a···O15	0.86	2.27	2.935 (6)	135
N8—H8a···O34^v^	0.86	2.21	2.957 (6)	145
N8—H8b···O23^iv^	0.86	2.23	2.840 (6)	128
N9—H9a···O35	0.86	2.40	2.992 (6)	126
N9—H9b···O24	0.86	2.10	2.846 (5)	145
N9—H9c···O19	0.86	2.06	2.895 (5)	165
N10—H10a···O11	0.86	2.04	2.879 (6)	165
N10—H10b···O30^viii^	0.86	2.39	3.176 (6)	153
N10—H10c···O19	0.86	2.05	2.884 (6)	164

Symmetry codes: (i) −*x*, −*y*+1, −*z*+1; (ii) −*x*, −*y*+2, −*z*+1; (iii) *x*−1, *y*+1, *z*; (iv) −*x*+1, −*y*+1, −*z*+1; (v) *x*+1, *y*, *z*; (vi) −*x*+1, −*y*+1, −*z*+2; (vii) *x*, *y*+1, *z*; (viii) −*x*, −*y*+1, −*z*+2.
